# Characterization of N-Glycan Structures on the Surface of Mature Dengue 2 Virus Derived from Insect Cells

**DOI:** 10.1371/journal.pone.0132122

**Published:** 2015-07-24

**Authors:** Y Lei, H Yu, Y Dong, J Yang, W Ye, Y Wang, W Chen, Z Jia, Z Xu, Z Li, F Zhang

**Affiliations:** 1 Department of Microbiology, School of Preclinical Medicine, The Fourth Military Medical University, 169 Changle Xi Road, Xian, Shaanxi 710032 China; 2 Laboratory for Functional Glycomics, College of Life Sciences, Northwest University, 229 Taibai Beilu, Xi’an 710069, China; 3 Department of Infectious Diseases and Center of liver Diseases, Tangdu Hospital, the Fourth Military Medical University, 569 Xinsi Road, Xi’an 710038, China; University of Missouri, UNITED STATES

## Abstract

DENV envelope glycoprotein (E) is responsible for interacting with host cell receptors and is the main target for the development of a dengue vaccine based on an induction of neutralizing antibodies. It is well known that DENV E glycoprotein has two potential N-linked glycosylation sites at Asn67 and Asn153. The N-glycans of E glycoprotein have been shown to influence the proper folding of the protein, its cellular localization, its interactions with receptors and its immunogenicity. However, the precise structures of the N-glycans that are attached to E glycoprotein remain elusive, although the crystal structure of DENV E has been determined. This study characterized the structures of envelope protein N-linked glycans on mature DENV-2 particles derived from insect cells via an integrated method that used both lectin microarray and MALDI-TOF-MS. By combining these methods, a high heterogeneity of DENV N-glycans was found. Five types of N-glycan were identified on DENV-2, including mannose, GalNAc, GlcNAc, fucose and sialic acid; high mannose-type N-linked oligosaccharides and the galactosylation of N-glycans were the major structures that were found. Furthermore, a complex between a glycan on DENV and the carbohydrate recognition domain (CRD) of DC-SIGN was mimicked with computational docking experiments. For the first time, this study provides a comprehensive understanding of the N-linked glycan profile of whole DENV-2 particles derived from insect cells.

## Introduction

Dengue virus (DENV) is the most important arthropod-borne human pathogen that is transmitted by the *Aedes aegypti* mosquito in tropical and subtropical countries[1]. During the last few decades, the incidence of dengue fever (DF) has increased dramatically. It is estimated that nearly 50to 100 million DF cases occur annually worldwide, including 500,000 dengue hemorrhagic fever (DHF) cases[2]. There are four genetically related DENV serotypes, and it is believed that DHF may result from secondary infection with different virus serotypes in which antibody-mediated disease enhancement (ADE) is involved[3]. This feature makes developing a DENV vaccine very difficult because an effective vaccine must successfully protect people against all four virus serotypes. Therefore, understanding the structure and function of the viral surface glycoprotein can be helpful in designing potent immunogens that safely protect against disease[4].

DENV is a positive-sense, single-stranded RNA virus whose genome encodes a polyprotein that is processed to produce three structural proteins, including capsid (C), premembrane/membrane (prM/M) and envelope (E), and seven nonstructural (NS) proteins, including NS1, NS2A, NS2B, NS3, NS4A, NS4B and NS5[5]. The E glycoprotein is the major component of the virion surface and interacts with receptors present on host cell surfaces, leading to endocytosis of the virus particle. E glycoprotein also induces humoral immune responses in which neutralizing antibodies can reduce the viral load[6]. Therefore, most vaccines being developed against DENV are based on the stimulation of immune responses towards the E glycoprotein. Although the crystal structure of DENV E glycoprotein has been determined, the glycans that are attached to the E glycoprotein are not fully understood[7]. Generally, N-glycans on the E glycoprotein have been shown to influence the proper folding of the protein, its interactions with receptors and its immunogenicity. [8]. It is well known that DENV E glycoprotein has two potential N-linked glycosylation sites at Asn67 and Asn153[9]. Smith & Wright first reported that the sugars that are added to the E protein are heterogeneous in structure and composition[10]. Subsequently, many works have shown that mosquito-derived DENV glycoproteins are a mix of high-mannose and paucimannose glycans[8, 11]. Dendritic cells(DC) cells in the skin are believed to be primary target cells of DENV during viral pathogenesis in the human body. It was recently shown that the high-mannose glycans on mosquito-derived DENV particles efficiently interact with DC-specific intercellular adhesion molecule3-grabbing non-integrin (DC-SIGN), rendering the virus able to enter immature DCin the skin following a bite of an infected mosquito[8]. In addition, a cryoelectron microscopy reconstruction of DENV complexed with the carbohydrate-binding domain of DC-SIGN has shown an interaction of a lectin with the N-glycan at Asn-67[12]. More recently, mimicking the cluster presentation of glycans on the virus surface has shown to be a promising strategy for designing carbohydrate-based antiviral agents. For example, oligomannosides (mannoGNPs) of gp120 high mannose-type glycans have been prepared and were able to inhibit DC-SIGN-mediated HIV-1 infection[13]. Therefore, obtaining detailed characteristics of carbohydrate structural information related to insect-derived DENV envelope proteins is helpful toward understanding interactions between the viral glycoprotein and host receptors, as well as for the development of E-related treatments for DENV infection. However, the defined carbohydrate structure on the surface of insect-derived DENV E glycoprotein that mediates attachment to its cell receptor remains elusive.

It remains an analytical challenge to elucidate the exact structures of the glycans that are attached to the glycoprotein surface due to the inherent heterogeneity of glycans at any given glycosylation site and because of variable glycosylation site utilization[14]. Recent technological advances in analytical methodologies have provided effective means of resolving this inherent heterogeneity and diversity of N-linked glycans. Among the analytical methodologies that are routinely employed in the analysis of protein glycosylation, lectin microarray has been considered a key tool for profiling sugar moieties on glycoproteins because lectins selectively recognize specific oligosaccharide epitopes[15]. Structural analysis of N-linked glycans released from peptide N-glycosidase F (PNGaseF) digestion can be resolved by MALDI-TOF-MS (Matrix-Assisted Laser Desorption/Ionization Time-of-Flight Mass Spectrometry) or by use of PGC (porous graphitized carbon) liquid chromatography in combination with on-line ESI MS[16].

Here, we report a comprehensive and detailed mapping of the carbohydrate profile of the surface of mature DENV-2 derived from insect cells. Using an integrated strategy based on lectin microarray and MALDI-TOF-MS, it was found that high mannose-type N-linked oligosaccharides and galactosylated N-glycans were the major glycan structures found on the DENV-2 E protein. Our results complement existing crystallography studies of the DENV-2 E protein and provide detailed information on the N-linked sugars of the DENV-2 surface for the first time. This integrated strategy of analyzing N-glycan structures on the surface of a whole virus can be applied to the other viruses.

## Materials and Methods

### Cell lines and virus strains

C6/36 *Aedes albopictus* cells (ATCCCRL-1660) were cultured in RPMI-1640 media supplemented with 1% L-glutamine, 1% penicillin/streptomycin, 1% non-essential amino acids and 10% fetal bovine serum (FBS; Gibco/Invitrogen),adjusted to pH6.8, at 28°C in 5% CO_2_. The DENV strain used in this study was DENV-2 16681.

**Fig 1:**
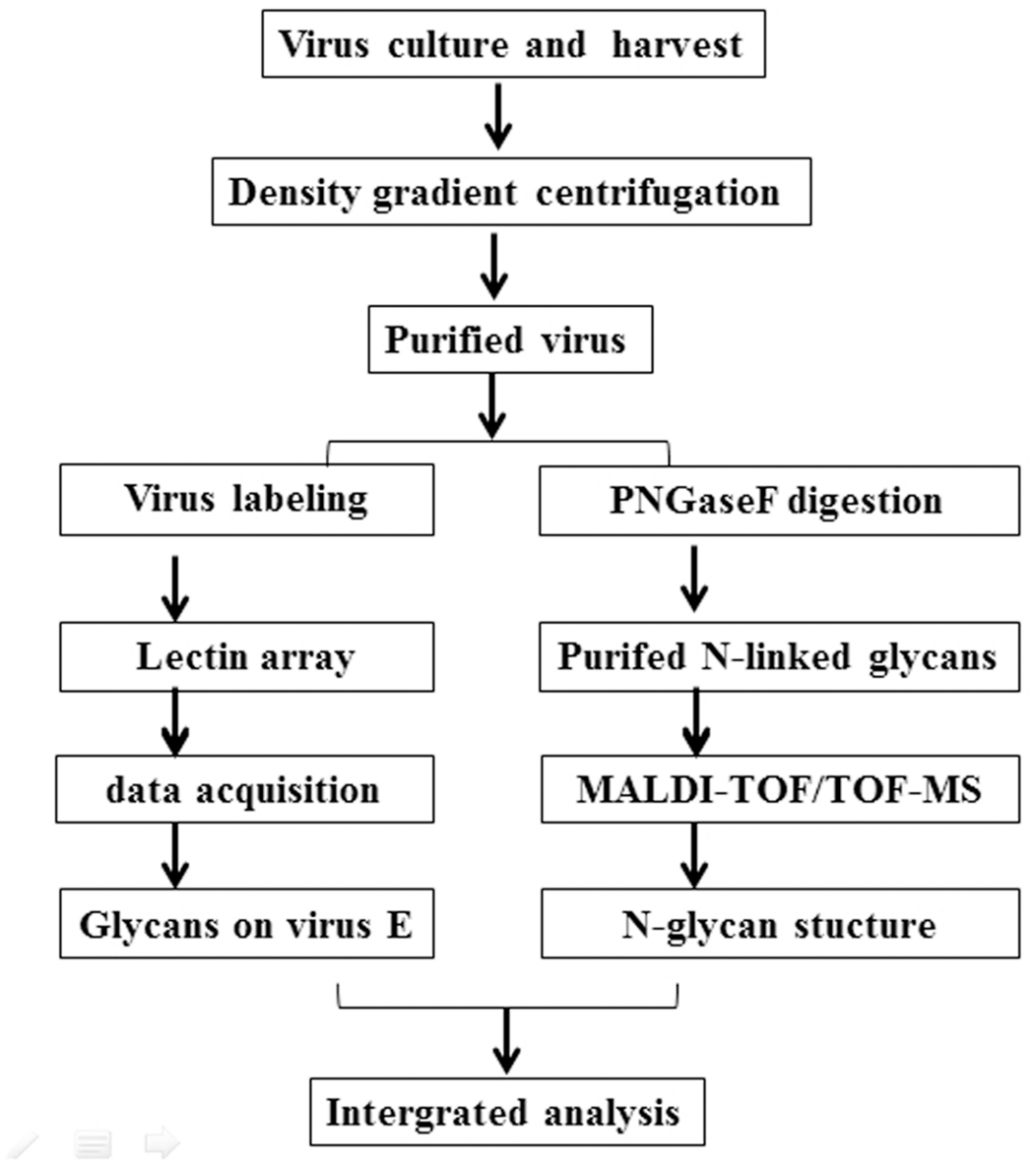
Workflow including virus purification, lectin array and MALDI-TOF/TOF-MS analysis.

The workflow from virus preparation to N-glycan analysis is represented in Fig 1.

### Virus propagation and purification of mature DENV-2 virions

To generate insect cell-derived DENV-2 virus, DENV-2 stocks were added to 60% confluent C6/36 mosquito cells at a multiplicity of infection (MOI) of 0.1 for 2 hours at 37°C, and the inoculant was replaced by RPMI-1640media containing 10% fetal bovine serum (FBS). At 24 hours post infection, the media was replaced with fresh RPMI-1640 containing 2% FBS at pH6.8. After 5–7 days, media was harvested from cells when a cytopathic effect became apparent and was clarified by centrifugation at 3000 g for 30 min. The virus suspension was mixed with 8% PEG 8000 at equal volume overnight at 4°C and centrifuged at 10,000 g for 1 h to pellet the virus. The pelleted virus was resuspended in PBS and loaded onto a 15–60% (v/v) continuous sucrose gradient at 110,000 g for 17 hr at 4°C. The fraction containing purified virus was concentrated, and buffer was exchanged into NTE buffer containing 12 mM Tris at pH 8, 120 mM NaCl, and 1 mM EDTA using an Amicon Pro100 kD MWCO concentrator (Millipore). The yield and purity of dengue virus were examined using SDS-PAGE followed by Coomassie blue staining. The purified DENV-2 virus was observed by electron microscopy after negative staining as described elsewhere.

### Culture and infection of DENV-2 on MDDCs from human PBMCs

White buffy coat preparations from healthy donors were obtained from the Blood Bank in Xi’jing Hospital, China. Human peripheral blood mononuclear cells (PBMCs) were first isolated by density gradient centrifugation on Ficoll-Hypaque (Pharmacia, Uppsala, Sweden). PBMCs were gently rotated at 4°C to form aggregates of monocytes. After sedimentation of the monocytes, the pellet was grown in RPMI 1640 culture media supplemented with or without 25 ng/ml IL-4 and50 ng/ml GM-CSF (both from R&D Systems, Minneapolis, MN, USA). At day 3, half of the culture media was exchanged with fresh media containing IL-4 and GM-CSF. After 5-7days, IL-4and GM-CSF were used to differentiate monocytes into immature MDDCs. Immature MDDCs were infected with DENV-2 at an MOI = 0.1 for 48 hrs.

### Immunofluorescence assay

The infected cells were fixed in 4% paraformaldehyde for 15 min at room temperature and permeabilized with 0.25% Triton X-100 for 10 min at room temperature. After three washes with PBST, the cells were incubated with PBS containing 3% bovine serum albumin (BSA) (Sigma-Aldrich, St. Louis, MO, USA) in PBS. The cells were then stained with anti-DENV mAb (clone 4G2) followed by incubation with cy3-conjugated goat-anti-mouse IgG(Invitrogen). Nuclei were stained with DAPI (4', 6-diamidino-2-phenylindole).

### Glycosidase digestion of envelope protein of purified DENV-2

To deglycosylate the purified DENV-2 particles that were produced by insect cells, 100 μg of DENV-2 particles were denatured by heating at 100°C for 10 min in the presence of 0.5% SDS and 40 mM dithiothreitol. After cooling, 1 μl of 500,000 U/ml PNGase F (Takara, Japan) was added, and the mixture was incubated in 5× reaction buffer (Takara, Japan) at 37°C for 1 h. Subsequently, the samples were subjected to SDS-PAGE and western blot analyses using anti-DENV hyperimmune mouse serum as described elsewhere.

### Fabrication of lectin microarray

As a high-throughput glycomics technology, lectin array was utilized to investigate the glycopatterns of dengue virus in this study. As previously described[17, 18], briefly, 37 lyophilized lectins (purchased from Vector Laboratories, Sigma-Aldrich, and Calbiochem), which included both N- and O-linked glycans, were fabricated into a lectin array. Each lectin was dissolved at a concentration of 1 mg/mL in manufacturer's recommended buffer containing 1 mmol/L appropriate monosaccharide and spotted onto epoxysilane-coated slides (homemade) via a Capital Smart Arrayer(CapitalBio, Beijing) at 70% humidity. Three replication spots per lectin were printed in each block, and 3 blocks were printed per slide. Printed slides were incubated in a humidified box overnight at room temperature and dried for 2 h in a vacuum drier at 37°C for immobilization. Following this, the lectin arrays were stored at 4°Cprotected from light when not being used immediately. For detailed information of the lectin array, please refer to S1 Table.

### DENV-2 virus labeling and data acquisition

The concentration of purified DENV-2 was determined using a BCA assay (Pierce). Cy3 mono-Reactive Dye (GE healthcare) was dissolved in DMSO and incubated for 1 h in darkness. Subsequently, 100μg purified virus was mixed with Cy3 Dye in an alkaline solution of 0.1 mol/L Na_2_CO_3_ (pH 9.3) and incubated for 3 h at room temperature for protein labeling. After incubation, the labeled virus was purified by Sephadex G-25 columns (GE healthcare) following manufacturer's specifications.

Unreacted epoxy groups were blocked for 1 h with blocking solution (2% (w/v) BSA in 10 mM PBS, pH 7.4) in a rotisserie oven at 25°C. After blocking, the slides were washed with PBST and PBS twice to remove uncombined lectins and were then dried by centrifugation. A total of 4μg Cy3-labeled virus was mixed with incubation solution containing 2%(w/v) BSA, 7.5%(w/v) glycine and 0.05%(v/v) Tween-20 in 10 mM PBS (pH7.4) and allowed to incubate with blocked lectin arrays. The incubation was performed at 37°C for 3 h in a rotisserie oven (Robins Scientific) set at 4 rpm. The slides were washed with PBST (10 mM PBS (pH7.4) with 0.05%(v/v) Tween-20) twice for 5 min each and then PBS for 5 min, after which they were dried by centrifugation. The lectin arrays were scanned with a Genepix 4000B confocal microarray scanner (Axon Instruments, USA) in the Cy3 channel, and the fluorescence intensity of each spot was extracted by Genepix 6.0 software (Axon Instruments, Inc. USA). To eliminate non-specific binding signals, the spots with SNR values (signal-to-noise ratio) of less than 3 were removed from the data. The net fluorescence intensity value for each spot was calculated by subtracting the median of the background value from the median of the F532 raw signal intensity value. The net intensity value of each lectin was represented by the mean ± SD.

### Release and Purification of N-linked glycans from mature DENV-2

First, purified mature DENV-2 was lysed with 1% Triton X-100 for 2 h at 37°C, after which detergent-solubilized viral proteins were transferred into a centrifugal filter device(Amicon Ultra-0.5 3KD device, Millipore). The filters were centrifuged at 12,000g for 15 min, and buffer was exchanged with 40 mmol/L NH_4_CO_3_; this procedure was repeated several times until Triton X-100 was completely eliminated. Subsequently, a solution of DENV-2 was denatured with 8 M urea, 10 mM DTT, and 10 mM IAM (Sigma-Aldrich) and then centrifuged. The ultrafiltration unit was transferred into a new collection tube, and DENV-2 proteins were digested with Sequencing Grade Trypsin (Promega) overnight at 37°C in a water bath. After incubation, the resultant polypeptides were collected by centrifugation. Following this, the polypeptides were further digested with PNGase F (New England BioLabs; Ipswich, MA, USA) overnight at 37°C. The released N-linked glycans were collected and desalted by Sepharose4B(Sigma-Aldrich) according to the manufacturer’s protocol, and eluted N-linked glycans were collected and lyophilized.

### Mass spectrometry analysis of N-linked glycans of mature DENV-2

Mass spectrometric analysis was carried out on a MALDI-TOF/TOF-MS (UltrafleXtreme, Bruker Daltonics; Bremen, Germany) as described previously[19]. Briefly, lyophilized N-linked glycans of DENV were resuspended in10 μL methanol/H_2_O (1:1, v/v) and 1 μL of the mixture was spotted onto an MTP Anchor Chip sample target and air-dried. A1 μL aliquot of matrix solution (DHB was dissolved in methanol/H_2_O (1:1, v/v) at a concentration of 20 mg/mL) was spotted to recrystallize the glycans. Measurements were taken in positive-ion mode, and *m/z*data were annotated by GlycoWorkbench software (http://code.google.com/p/glycoworkbench/). Relative intensity was analyzed and generated by Flex Analysis software (Bruker Daltonics) based on MALDI-TOF-MS intensity.

### Docking analysis of DC-SIGN and glycan receptor complexes

To compare the interactions between DC-SIGNs and glycan receptors, six representative glycans were designed and constructed to contain either a natural mannose glycan receptor (Hex3HexNAc2,NR), a high-mannose glycan (Hex9HexNAc2,HM), a hybrid type N-glycan (Hex7HexNAc4,HY), a galactosylated glycan (Fuc1Hex6HexNAc5,GS) orasialylated complex-type glycan (NeuAc1Fuc2Hex5H,SC); additionally, a 6-glucose-hexose (6G) was used as a negative quality control. All of the potential receptors were constructed with the SWEET2 program and optimized using an MM3 force field[20].

The DC-SIGN model was obtained from the PDB (Protein data bank;PDB ID: 1K9J)and was used for flexible docking. This model was docked with 6 receptors that were selected from the above by turns. Flexible docking was performed using AutoDock 4.2,in which the grid spacing was set to 0.375 Å, and each grid map consisted of 80×80×80 grid points in 3-D. After each docking analysis was performed, the grid center coordinates were reset as the mean coordinates of the receptor-binding domain. The glycosidic linkages from the receptors acted as flexible linkages and were allowed to engage in rotation in twenty-degree increments, together with crucial amino-acid residues, such as Asn36, Glu366, Ser363 and so on. In addition, any other options were set to their default values. A total of 100 docking runs were adopted using a Lamarckian genetic algorithm to calculate binding energies, and the most probable docking conformation was generated during each docking experiment. In the end, the results were analyzed by means of cluster analysis of the largest cluster. Both complementary analysis and flexible docking calculations (AutoDock4.2) were performed using a Windows XP workstation.

## Results

### Purified mature DENV-2 derived from insect cells can efficiently infect MDDCs

**Fig 2:**
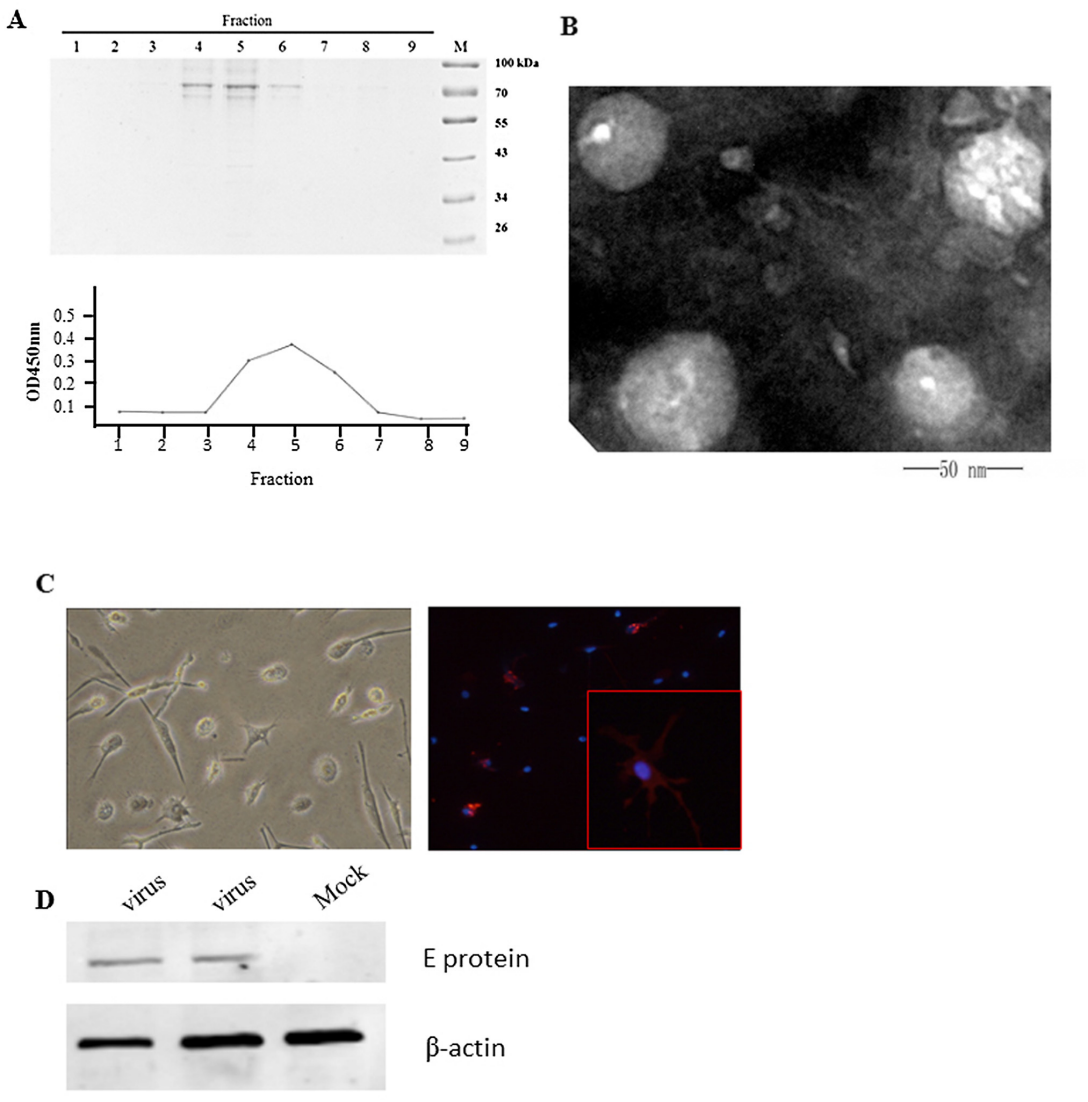
Purification of DENV-2 particles from insect cells and infection of MDDC. (A) DENV-2 virus was concentrated and purified via sucrose density gradient centrifugation. Fractions from the gradients were analyzed by SDS-PAGE, and dengue virus was mainly found in fractions4, 5 and 6 after centrifugation (upper panel). Double anti-DENV-2 antibody ELISA results were consistent with electrophoresis(lower panel).(B) Purified mature DENV-2 virions were negative stained and observed via transmission electron microscopy. Mature dengue virions were approximately 50 nm in diameter, surrounded by lipid bilayers, and had‘‘smooth” regions on their outer membranes. (C) Monocytes isolated from PBMCs were treated with 25 ng/ml IL-4 and 50 ng/ml GM-CSF for 7 days and infected with DENV-2 at an MOI = 0.1 for 48 hours. Two days after infection, the cells were permeabilized and analyzed for DENV-2 E protein expression using a 4G2 antibody. Nuclei were stained with DAPI (blue). (D) The expression of DENV-2 E was demonstrated by western blotting using anti-DENV-2 hyperimmune serum.

Previous studies have shown that a heterogeneous population of virions that vary in maturation state are produced from DENV-infected cells[21]. It is well known that an incomplete cleavage of the envelope glycoprotein prM generates a mixture of mature (prM-less) and immature extracellular particles(prM-containing) during the dengue virus life cycle[22]. In vitro infections with immature and mature dengue viruses have shown that fully immature particles are non-infectious because they block virus-receptor interactions and membrane fusion activity via the prM protein. To characterize the glycans that decorate the surfaces of mature dengue virions, we optimized culture conditions with RPMI-1640 media and created a sucrose density gradient centrifugation method to harvest mature dengue virus. As indicated in Fig 2A, SDS-PAGE shows that dengue virus was mainly in fractions 4, 5 and 6 after centrifugation (upper panel). Double anti-DENV-2 antibody ELISA results were consistent with the results from electrophoresis(lower panel). The fraction that contained the purified mature virions was harvested. It is known that immature DENV particles have mosaic structures with ‘‘spiky” regions and that mature virions have‘‘smooth” regions, as demonstrated by structural studies. Furthermore, mature DENV-2 virions were negative-stained and observed using transmission electron microscopy. Fig 2B shows that the mature dengue virions were approximately 50 nm in diameter and surrounded by a lipid bilayer and had‘‘smooth” regions on their outer membranes.

Immature dendritic cells (imDCs) are the main target cells for DENV replication after a bite of an infected mosquito[23]. To determine whether mature dengue virus can infect imDCs, we isolated PBMCs and differentiated them into imDCs as indicated in Fig 2C. IFA showed that imDCs was easily infected with mature DENV-2 virus. To further validate these results, western blot was performed using anti-DENV-2 hyperimmune serum. The results supported thatDENV-2 was replicated in imDCs (Fig 2D).

### Glycosylation of envelope proteins on the surfaces of DENV-2 particles

**Fig 3:**
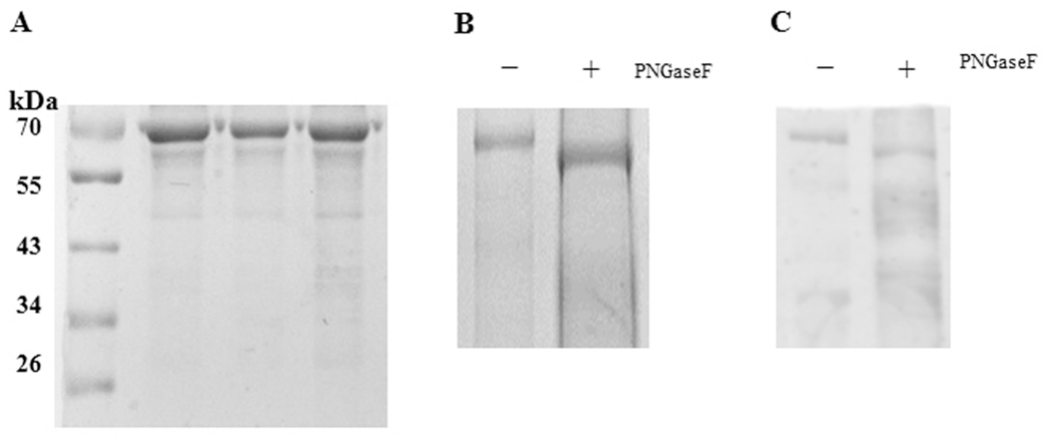
N-Linked glycosylation status of purified DENV-2 was analyzed by SDS-PAGE and WB after PNGase F treatment. (A) Purified virus was observed by SDS-PAGE. (B) Purified virus was digested with PNGaseF (+) or mock digested (-) and evaluated by SDS-PAGE. (C) Western blotting was performed to show the deglycosylation patterns of envelope proteins of DENV-2 virions using anti-DENV-2 hyperimmune serum.

To evaluate the glycosylation patterns of envelope proteins on purified DENV-2 produced by insect cells, a deglycosylation assay using N-endoglycosidase PNGase F digestion was performed. PNGase F removes all types of N-linked oligosaccharides from glycoproteins. Fig 3A & 3B show that without PNGase F treatment a 70kDa band was detected by SDS-PAGE, whereas with PNGase F treatment DENV-2 envelope bands were reduced to a 60kDa band. A similar deglycosylation pattern was observed in the envelope proteins of DENV-2 virions with western-blot (Fig 3C). Thus, these results revealed that the envelope proteins of DENV-2 virions, when produced by insect cells, posses a high degree of N-linked glycosylation.

### Lectin microarray

**Fig 4:**
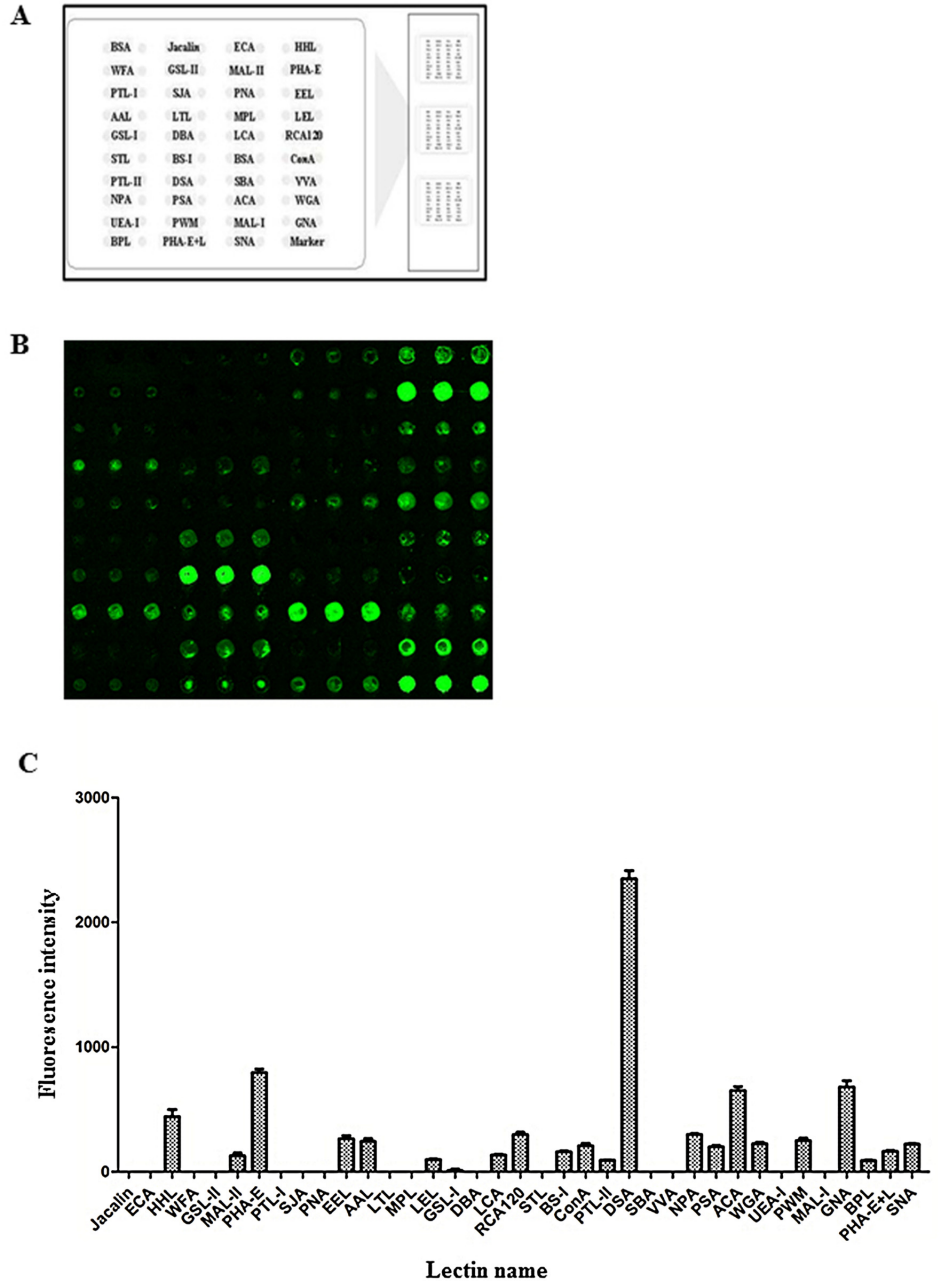
Glycan profiling of purified DENV-2 particles from insect cells by lectin microarrays. (A) The layout of the lectin microarray. A total of 37 lectins were dissolved in manufacturer's recommended buffer to a concentration of 1 mg/mL and spotted onto homemade, epoxysilane-coated slides according to aprotocol from Stealth micro spotting pins (SMP-10B). Each lectin was spotted in triplicate per block, and triplicate blocks were placed on one slide. Cy3-labeled BSA was spotted as a location marker, and BSA was used as a negative control. (B) A profile of Cy3-labeled DENV-2 virions derived from insect cells bound to the lectin microarrays. Fluorescent images were scanned in a 70% photomultiplier tube at a 100% laser power setting with a Genepix 4000B confocal scanner. A representative portion of a slide with three replicates of the lectin array is shown. (C) The relative fluorescence intensities of cy3-labeled DENV-2 binding to 37 lectins. A total of 21 lectins out of 37 showed positive binding signals.

**Fig 5:**
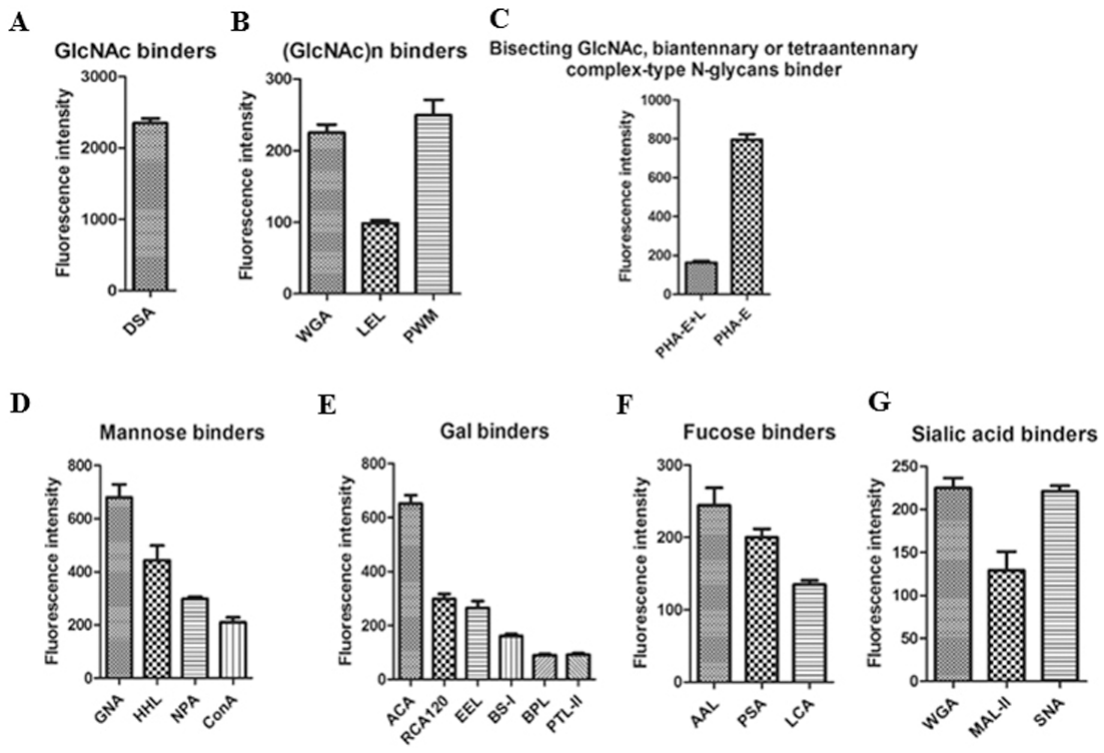
Relative expression levels of DENV-2 glycan binders by lectin microarray. The glycans binders were categorized into five types. (A) The GlcNAc binder DSA showed a stronger binding signal than the others. (B) (GlcNAc)n binders. (C) Bisecting and biantennary GlcNAc binders. (D) Mannose binders. (E) Gal binders. (F) Fucose binders. (G) Sialic acid binders.

To identify patterns of glycosylation on DENV, we performed an analysis using a lectin microarray with Cy3 labeled-DENV-2. Aresultant image of the incubationis shown in Fig 4A, and the fluorescent intensities of the assessed lectins are shown in Fig 4B. According to the results, 21 out of 37 lectins showed positive binding signals. Among these, the GlcNAc binder DSA showed a stronger binding signal than the others (see Fig 5A), which indicated that GlcNAc was a dominant glycopattern in DENV-2. Meanwhile, the binding signal produced by PHA-E was stronger than those produced by other lectins, except DSA, which indicated that glycopatterns inclusive of bisected GlcNAc and biantennary N-glycans are in high abundance on DENV-2 (shown in Fig 5C). The values of the binding signals of HHL and GNA, which were identified as high mannose, were also greater than 500, demonstrating that high mannose is an N-glycan component in DENV-2. According to the specificity of lectins, the resultant glycan profile of DENV provided the following information: (i) gal, α-gal structures exist in the glycan profile of DENV and are associated with signals from ACA, RCA120, EEL, BS-I, BPL and PTL-II (Fig 5D);(ii) a glycoform of high-mannose was detected in DENV, which was associated with signals from GNA, HHL NPA and ConA (Fig 5E);(iii) fucose content (especiallyfucoseα-1,6GlcNAc) was evident in the glycan profile of DENV in AAL, PSA and LCA (Fig 5F);(iv) sialic acid content (Sia2-3/6Gal, Multivalent Sia) also emerged in the glycan profile of DENV-2 and was evident by signals corresponding to MAL-II, SNA and WGA (Fig 5G).

### Analysis of PNGase F-Released Glycans from DENV

**Fig 6:**
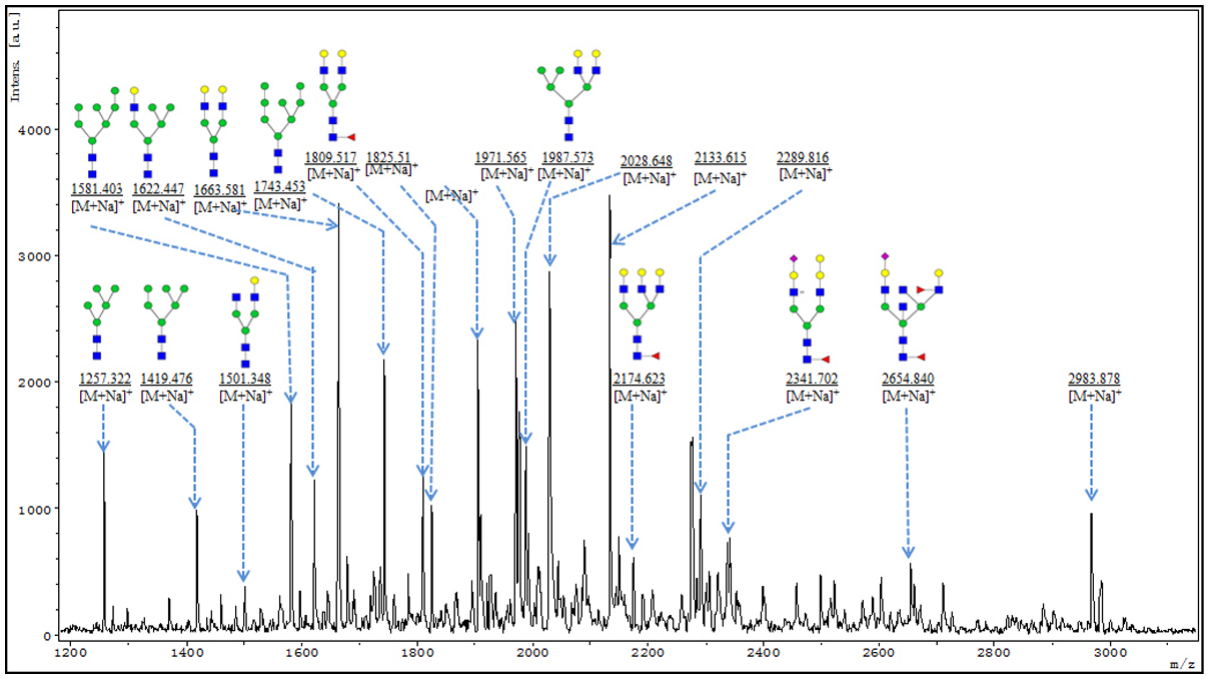
MALDI-TOF-MS spectra of N-glycans on purified mature DENV-2. N-glycans from purified mature DENV-2 virions were separated and desalted as described in M&M. Lyophilized N-glycans were dissolved in MW, and an aliquot of a mixture with DHB solution was spotted onto an MTP Anchor Chip sample target and air-dried. MALDI-TOF-MS was performed in positive-ion mode. Experiments were performed in biological triplicate, and representative N-glycan spectra are shown. Peaks (signal-to-noise ratio >5) were selected for relative proportion analysis. Detailed structures were analyzed using Glyco Work bench software. Proposed structures are indicated by m/z values.

**Fig 7:**
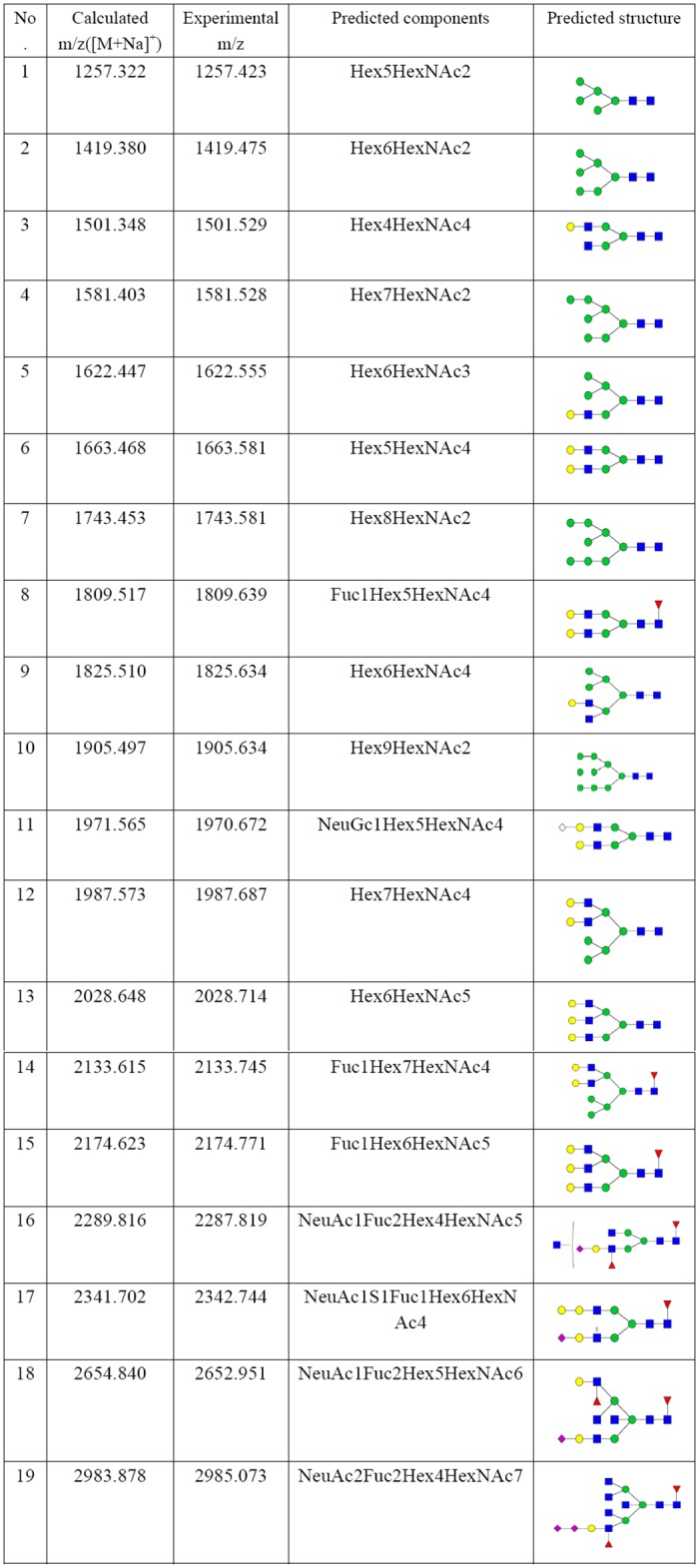
Predicted structures and their molecular ions in MALDI Spectra of N-Glycans from purified mature DENV-2 virions.

**Fig 8:**
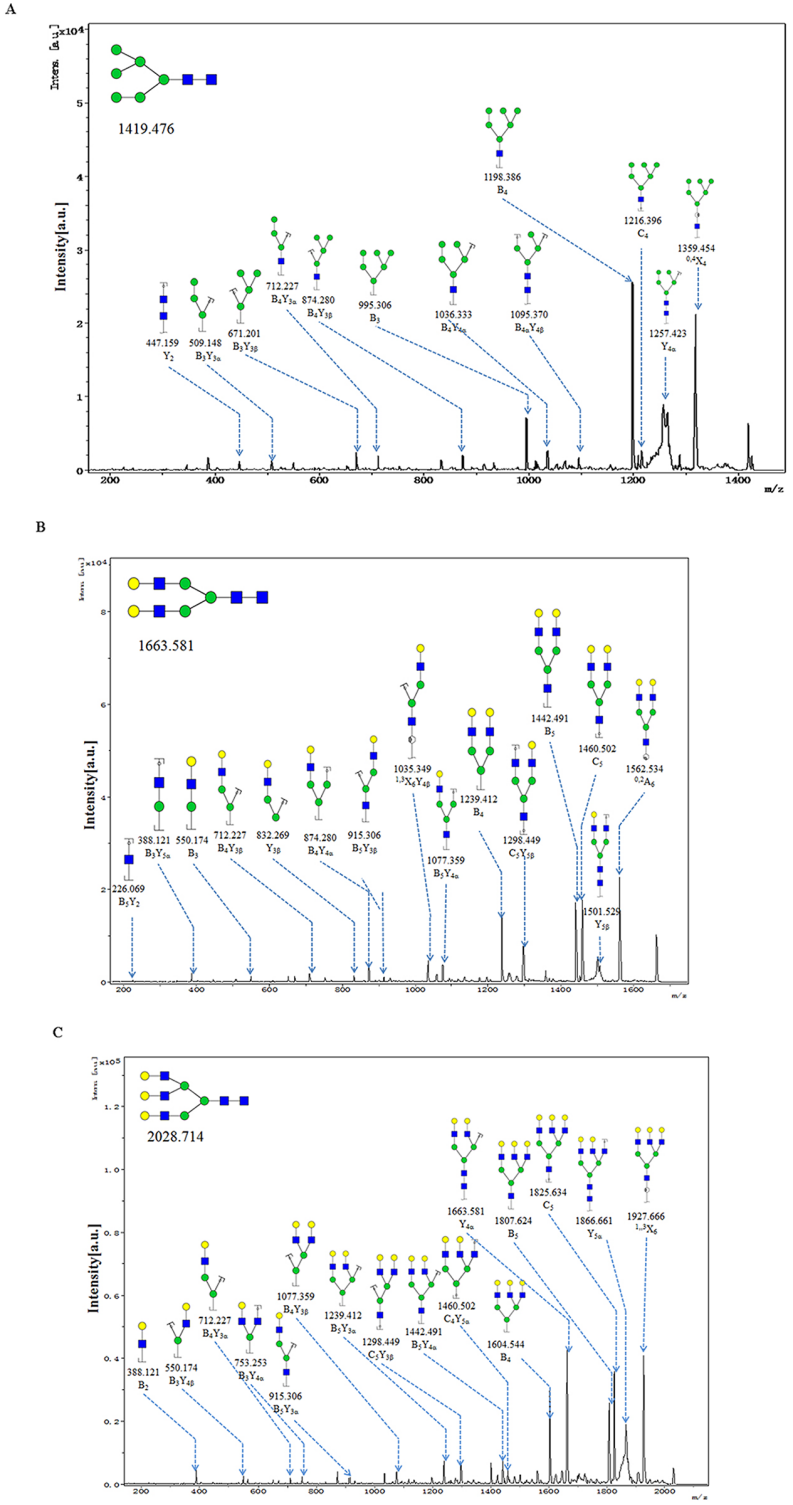
MALDI-TOF/TOF-MS/MS analysis of N-glycan precursor ions in MS spectra. Precursor ions were subjected to MS/MS analysis to obtain cleavages, including glycosidic cleavages and cross-ring cleavages. Structures of cleavage ions and m/z values are shown in tandem mass spectra. Three major N-glycan peaks are indicated: (A) m/z 1419.476, (B) m/z 1663.581, and (C)m/z 2028.714.

MALDI-TOF/TOF-MS/MS was performed to obtain detailed information regarding substitutions and branching patterns of the studied monosaccharide constituents. To investigate the exact pattern of N-linked oligosaccharides on mature DENV-2 virions, MALDI-TOF-MS/MS analysis was employed. MALDI-TOF-MS spectra of N-linked oligosaccharides on DENV-2 with signal-to-noise ratios >4 were annotated using Glyco Workbench software (Fig 6). DENV showed 19 distinct m/z N-glycans, and hybridtype-N-glycans made up an important class of glycans isolated from DENV, such as the N-glycans at m/z1622.4, 1825.6, 1987.6 and 2133.6. Detailed information on these predicted N-glycans are shown in Fig 7. More remarkably,the hybrid type-N-glycan at 2133 m/z (Fuc1Hex7HexNAc4) is the most abundant N-glycan in DENV. In addition, several high-mannose type-glycans were identified in DENV, such as the species at m/z 1257.3, 1419.4, 1581.4, 1743.5 and 1905.5, which are shown in Fig 6. When we determined the N-glycan composition of DENV by MALDI-TOF-MS, we observed that galactosylated N-glycans dominated the N-linked oligosaccharides (15 of 19 N-glycans were galactosylated). MALDI-TOF-MS analysis also exhibited that some N-glycans were further modified with fucose and sialic acid; N-glycans at m/z 2133.6, 2174.6, 2289.8, 2341.7, 2654.8 and 2983.9 were modified with fucose moieties linked to either internal or external HexNAc residues (shownin Fig 6). Moreover, N-glycans at m/z 2289.8, 2341.7, 2654.8 and 2983.9 had terminal sialic acids (shown in Fig 6). To obtain detailed information on the N-linked oligosaccharides of DENV, the peaks that were detected in the N-glycan spectra were subjected to MALDI MS/MS analysis. MS/MS spectra of precursor ions at m/z 1419.380, 1663.468 and 2028.648 are shown in Fig 8A-8D. An oligosaccharide with mannose branches was revealed by fragment ions B_3_Y_3β_ (671.201) and B_4α_Y_4β_(1095.370)at m/z 1419.476(shown in Fig 8A). The existence of GalNAc residues in the N-glycan profile of DENV was identified byB_3_Y_5α_(388.121) at m/z 1663.581 and by B_3_Y_4α_(550.174) and Y_4α_(1663.581) at m/z 2028.714(shown in Fig 8B and 8C). In conclusion, MS profiling in combination with tandem mass spectrometry provided detailed information on the N-glycan profile of DENV.

### Molecular docking analysis between sugar carbohydrates on DENV-2 virions and DC-SIGN

**Fig 9:**
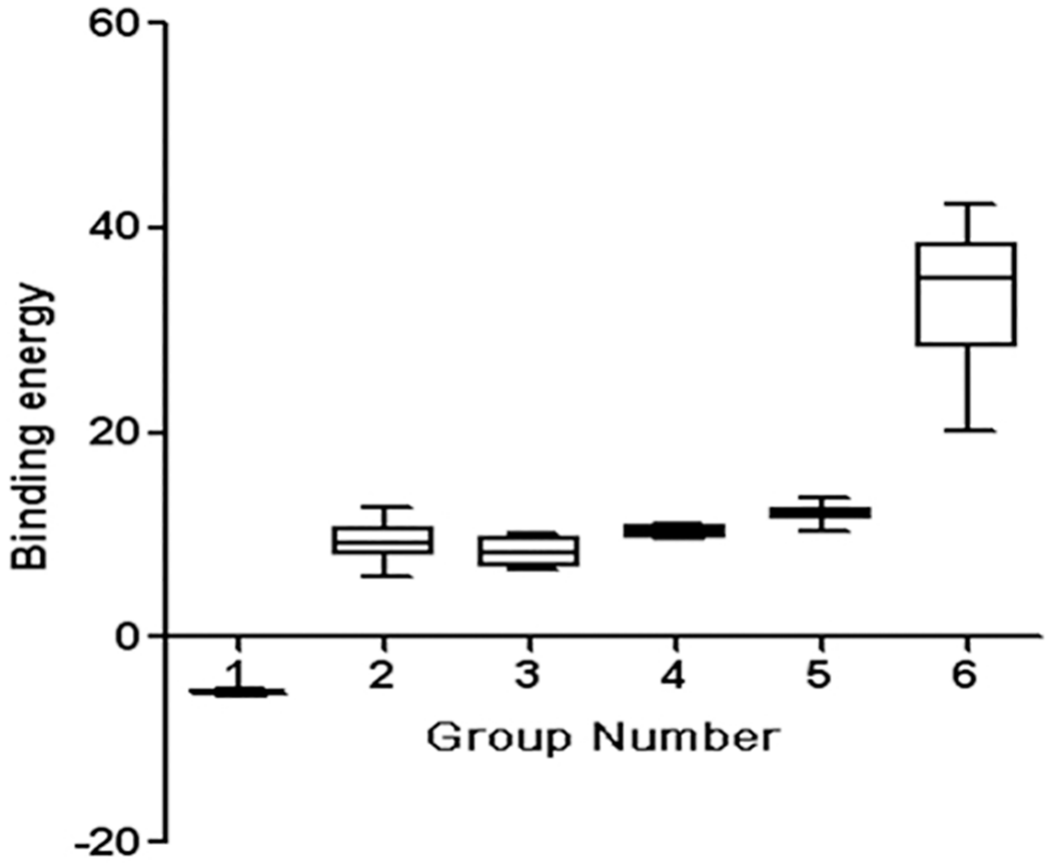
Binding energies between DC-SIGN and selected glycans on the DENV-2 virus surface as predicted by mass spectra. The 1–6 groups represent binding energies between DC-SIGN and a natural mannose glycan receptor (Hex3HexNAc2,NR), a high-mannose glycan (Hex9HexNAc2,HM), a hybridtype-N-glycan (Hex7HexNAc4,HY), a galactosylated glycan (Fuc1Hex6HexNAc5,GS) and asialylated complex type-Glycan (NeuAc1Fuc2Hex5H,SC), as well as a 6-glucose-hexose (6G).

DC cells are the main target cell type for dengue virus infection. An interaction between glycan moieties on the dengue virus E glycoprotein and the receptor molecule Dendritic Cell-Specific Intercellular adhesion molecule-3-Grabbing Non-integrin (DC-SIGN) facilitate virus-cell binding[8]. To define putative carbohydrate ligands binding to the crystal structure of DC-SIGN, 4 glycans were chosen for docking analysis based on our lectin array and MALDI-TOF-MS results. According to binding energies that were provided from output files, all DC-SIGN models possesse different affinities for 6 glycans (Fig 9). Interestingly, as a negative quality control, the binding energy from DC-SIGN-6G complexes presented the highest value, approximately +20 –+42 kcal/mol, which corresponded to a nonbinding property between DC-SIGN and glucose. In the docking assays, all DC-SIGNs and non-natural receptors presented relatively higher energies than the natural receptor, which indicated that these non-natural receptors were not specific to DC-SIGN. The binding energies that were obtained were mainly from one or two monosaccharide residues within the amino acid residues.

**Fig 10:**
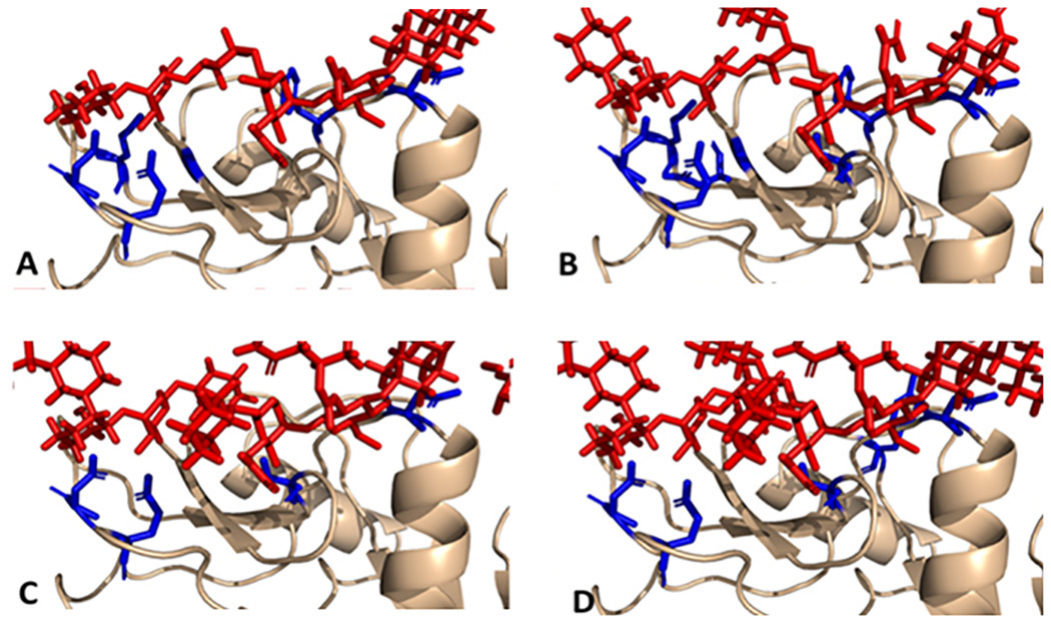
Docking analysis of DC-SIGN and glycan receptors. A-D represent docking conformations of DC-SIGN and HM, HY, GS and SC. As is shown, the identified interactions mainly originated between glycans and the amino acid residues shown in blue.

Hydrogen bonding (H-bond) and hydrophobic interactions are two of the forces that exist between glycan receptors and DC-SIGN. H-bonds play an essential role in stabilizing ligand-receptor complexes. It has been shown there are numerous polar groups in glycan receptors and in DC-SIGN that lead to hydrogen bond formation. Most of the persistent H-bonds were formed from hydroxyl groups onAsn36, Glu366, Ser363 and mannose (Fig 10). Many observable transient H-bonds were formed in the remainder of glycan receptors and in non-conserved residues around the glycan receptor.

## Discussion

In this study, we characterized the N-glycan profile of the surface of DENV-2 derived from insect cells. N-glycan structures were solved from purified DENV-2 virions by combining lectin microarray with MALDI-TOF-MS. Defining these DENV-2 envelope protein N-glycan structures is important in understanding how glycosylation affects the biological properties of the virus and for designing glycomimetic compounds as potential antiviral agents.

It has been shown that DENV E contains two N-linked glycosylation sites at Asn-67 and Asn-153[9]. N-linked glycans on DENV E proteins are involved in viral morphogenesis, infectivity, and tropism. Gamarnik etal. examined the relevance of each of the N-linked glycans on the dengue virus E protein by use of mutation of each site in the context of infectious viral particles and found that a lack of a carbohydrate at position 67 reduced the production of new infectious particles but did not affect viral entry or genome translation and replication[24]. Smith & Wright first reported that the sugars that are added to the DENV E protein are heterogeneous in structure and composition[10]. Subsequently, the glycans on the DENV envelope have been roughly characterized for several serotypes. However, there is no consensus as to what types and structures of sugars are added to the DENV E protein when it is produced in mosquito or mammalian cells. Recently, Kari Hacker et al.characterized structures of N-linked glycans on DENVs derived from insect and mammalian cells using glycosidase digestion assays and lectin blots[9]. They found that N-linked glycans on DENV virions derived from mammalian cells were a mix of high-mannose sugars and complex sugars, while a mix of high-mannose sugars and paucimannose sugars were found on mosquito-derived virions. Many studies of DENVN-linked glycans have shown that the heterogeneity of these glycans is very high and that far more than a dozen different types of glycans can be found. To date, no comprehensive studies have been performed to characterize the N-linked sugar structures at each of the potential glycosylation sites of DENV due to the unique analytical challenges that are presented by glycans and because of the overwhelming diversity of sugars in nature.

**Fig 11:**
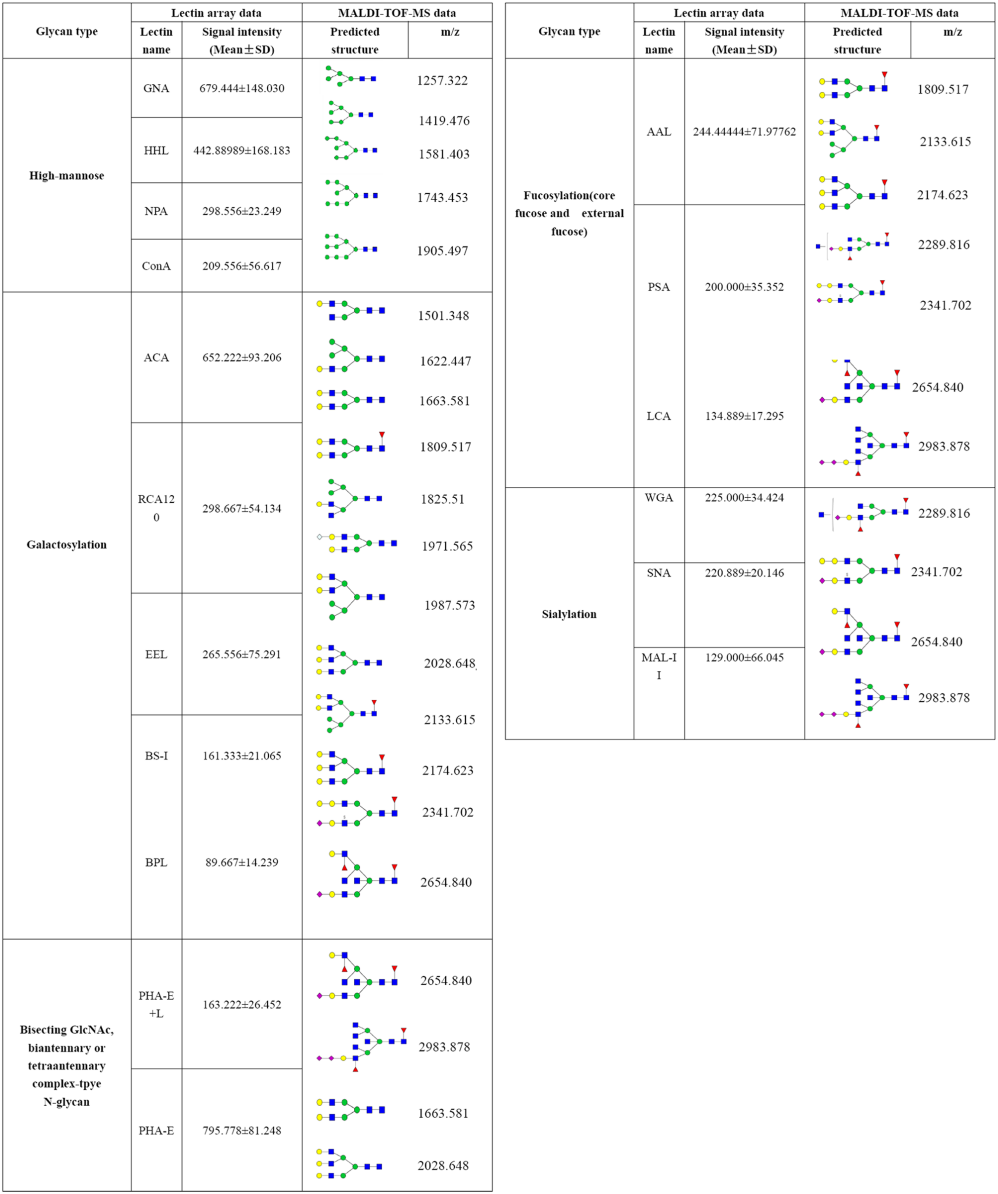
The proposed structural information of glycan on the surface of mature DENV-2 revealed by Mass Spectrometry and lectin microarray analysis.

It is known that lectin array has an advantage of obtaining detailed information about the structures of partial glycans, especially their linkage information. In addition, mass spectrometry is usually utilized to obtain information about the compositions of oligosaccharides[25]. To acquire precise structural information about the glycan profile of DENV, lectin array and mass spectrometry were employed in tandem in this study. When integrating the analyses of these results, precise structural information of the glycans on the surface of mature DENV-2 was obtained, which is shown in Fig 11. Consistent with a previous report, a high heterogeneity was found in the N-glycans on DENV [26]. Five types of N-glycans were identified on DENV-2, including mannose, GalNAc, GlcNAc, fucose and sialic acid, and high-mannose-type N-linked oligosaccharides and galactosylation of N-glycan were the major structures. According to the results of lectin arrays, the mannose branching/high-mannose-type N-glycan binders GNA, HHL, NPA and ConA showed positive binding signals, which indicated that mannose branching and high-mannose-type N-glycans exist in DENV. As a result of MS/MS, the fragment ions B_3_Y_3β_ (671.201) and B_4α_Y_4β_(1095.370)at m/z 1419.476 indicated the presence of oligosaccharides with mannose branches; furthermore, an MS/MS spectrum of a glycan at m/z 1419.476 revealed that high-mannose-type N-glycans exist in DENV. MS/MS analysis revealed ionsC_5_Y_5β_(1298.449) and Y_5β_(1501.529) at m/z 1663.581 andC_4_Y_5α_ (1460.502)and Y_5α_(1866.661) at m/z 2028.714, which were indicative of the presence of terminal GalNAc. Meanwhile, the binding signals of the gal binders ACA, RCA120, EEL, BS-I and BPL on lectin arrays also confirmed the existence of gal residues. The lectins PHA-E and PHA-E+L were detected via moderate binding signals, which revealed that bisecting GlcNAc, biantennary or tetraantennary complex-type N-glycans existed in the glycome of DENV. The ions at m/z 2654.840 and 2983.878 exhibited bisecting GlcNAc residues, and the predicted structures at m/z 1663.581, 2028.648 and 2983.878 in the MS spectrum verified the existence of biantennary or tetraantennary complex-type N-glycans on DENV. The results of MS also detected an N-glycan on DENV with an internal fucose residue (at m/z 1809.517, 2133.615, 2174.623,etc.) and an external fucose residue that was attached to GlcNAc (at m/z 2289.816, 2654.840 and 2983.878). Furthermore, according to the glycans recognized by the lectins AAL, PSA and LCA, it was indicated that α-fucose, especially α1,6-fucose (a core fucose that is recognized by PSA and LCA),was present on DENV. A sialic acid N-glycan was speculated to be present by MS spectra at m/z 2289.816, 2341.702, 2654.840 and 2983.878. Sialylation of DENV glycans was also identified by lectin array according to the binding signals of the lectins MAL-II (Siaα2,3-Gal binder), SNA (Siaα2,6-Gal binder) and WGA (multivalent sialic acid binder). It was noted that the intensity of the binding signal corresponding to SNA was almost twice that of MAL-II. This indicated the presence of sialic acids in DENV glycans. Moreover, the abundance of Siaα2,6-Gal was higher than Siaα2,3-Gal.

During DENV infection in vivo, it is well known that DC cells are the primary target cells of the virus[27]. It was recently shown that DENV productively infects DC cells by binding to DC-SIGN, which specifically recognizes and binds N-linked sugars on the E protein of DENV. DC-SIGN expressed in DC cells plays an important role in virus infection and transmission, making it an attractive target for interfering with dengue virus propagation[23]. However, details of the interaction between DC-SIGN and sugars on the DENVE glycoprotein have been unclear. DC-SIGN is composed of four domains, including a cytoplasmic domain, a transmembrane domain, seven to eight extracellular neck repeats responsible for its oligomerization and a carbohydrate recognition domain (CRD). A previous study demonstrated that the DC-SIGN CRD recognizes fucose- and high mannose-type N-glycan-containing blood group antigens[28]. Here, we have identified19 distinct m/z N-glycans on DENV-2,ofwhich high mannose-type N-linked oligosaccharides and the galactosylation of N-glycan were the major structures based on lectin array and MALDI-TOF-MS analyses. To define putative carbohydrate ligands that bind to the crystal structure of DC-SIGN, 4 glycans were selected based on binding energies between monosaccharide residues of putative DENV glycans and amino acid residues in the CRD using computational docking experiments. It was shown that all of the selected glycans can bind to DC-SIGN CRD and act as putative glycans on the surface of DENV-2 that are responsible for interacting with DC cells.

In this study, we obtained comprehensive and detailed mapping of the carbohydrate profile that corresponds to the surface of DENV-2 derived from insect cells. Mimicking sugars on the surface of DENV as a strategy for designing carbohydrate-based antiviral agents against DENV infection is underway. Therefore, a powerful alternative may be provided by glycomimetic compound-based inhibitors against dengue envelope protein to block the DC-SIGN–dengue interaction. Furthermore, the method described here is an effective way of analyzing N-glycan structures on the surfaces of whole virus particles.

## Supporting Information

S1 Table Sugar-binding specificities of the lectins. (DOCX)
