# Supplementary material for: Characterization of N-Glycan Structures on the Surface of Mature Dengue 2 Virus Derived from Insect Cells
Source: PLoS One. 2015 Jul 24;10(7):e0132122. doi: 10.1371/journal.pone.0132122 (PMC4514477; doi:10.1371/journal.pone.0132122)
Supplement: S1 Table — (DOCX) [file pone.0132122.s001.docx]

**S1 Table.** Sugar-binding specificities of the lectins

**Lectins**

Artocapus integrifolia (Jacalin), Erythrina cristagalli (ECA), Hippeastrum Hybrid Lectin (HHL), Wisteria Floribunda Lectin (WFA), Griffonia (Bandeiraea) Simplicifolia Lectin II (GSL-II), Maackia Amurensis Lectin II (MAL-II), Phaseolus vulgaris Agglutinin(E) (PHA-E), [Psophocarpus Tetragonolobus Lectin I](http://www.vectorlabs.com/products.asp?catID=240&locID=168) (PTL-I), [Sophora Japonica Agglutinin](http://www.vectorlabs.com/products.asp?catID=248&locID=168) (SJA), Peanut Agglutinin (PNA), [Euonymus Europaeus Lectin](http://www.vectorlabs.com/products.asp?catID=218&locID=168) (EEL), [Aleuria Aurantia Lectin](http://www.vectorlabs.com/products.asp?catID=207&locID=168) (AAL), [Lotus Tetragonolobus Lectin](http://www.vectorlabs.com/products.asp?catID=228&locID=168) (LTL), [Maclura Pomifera Lectin](http://www.vectorlabs.com/products.asp?catID=232&locID=168) (MPL), Lycopersicon Esculentum (Tomato) Lectin (LEL), [Griffonia (Bandeiraea) Simplicifolia Lectin I](http://www.vectorlabs.com/products.asp?catID=222&locID=168) (GSL-I), Dolichos Biflorus Agglutinin (DBA), [Lens Culinaris Agglutinin](http://www.vectorlabs.com/products.asp?catID=227&locID=168) (LCA), [Ricinus Communis Agglutinin I](http://www.vectorlabs.com/products.asp?catID=242&locID=168) (RCA120), [Solanum Tuberosum (Potato) Lectin](http://www.vectorlabs.com/products.asp?catID=247&locID=168) (STL), Bandeiraea simplicifolia (BS-I), Canavalia ensiformis (ConA), [Psophocarpus Tetragonolobus Lectin II](http://www.vectorlabs.com/products.asp?catID=241&locID=168) (PTL-II), Datura stramonium (DSA), Soybean Agglutinin (SBA), Vicia Villosa Lectin (VVA), Narcissus Pseudonarcissus Lectin (NPL), Pisum Sativum Agglutinin (PSA), Amaranthus caudatus (ACA), Triticum vulgaris (WGA), Ulex Europaeus Agglutinin I (UEA-I), Phytolacca americana (PWM), [Maackia Amurensis Lectin I](http://www.vectorlabs.com/products.asp?catID=230&locID=168) (MAL-I), Galanthus nivalis (GNA), [Bauhinia Purpurea Lectin](http://www.vectorlabs.com/products.asp?catID=210&locID=168) (BPL), Phaseolus vulgaris Agglutinin (E+L) (PHA-E+L) and Sambucus Nigra Lectin (SNA) were purchased from vector laboratories, Sigma-Aldrich and Calbiochem (Merck), respectively. The detail information was showed in S1 Table.

| Lectin | Specificity | Print  monosaccharide | Supplied by |  |
| --- | --- | --- | --- | --- |
| Jacalin | Galβ1-3GalNAcα-Ser/Thr(T), GalNAcα-Ser/Thr(Tn), GlcNAcβ1-3-GalNAcα-Ser/Thr(Core3), sialyl-T(ST). not bind to Core2, Core6, and sialyl-Tn (STn) | Galactose | Vector |  |
| ECA | Galβ-1,4GlcNAc (type II), Galβ1-3GlcNAc (type I) | Galactose | Vector |  |
| HHL | High-Mannose, Manα1-3Man, Manα1-6Man, Man5-GlcNAc2-Asn | Mannose | Vector |  |
| WFA | terminating in GalNAcα/β1-3/6Gal | GalNAc | Vector |  |
| GSL-II | GlcNAc and agalactosylated tri/tetra antennary glycans | GlcNAc | Vector |  |
| MAL-II | Siaα2-3Galβ1-4Glc(NAc)/Glc, Siaα2-3Gal, Siaα2-3, Siaα2-3GalNAc |  | Vector |  |
| PHA-E | Bisecting GlcNAc, biantennary complex-type N-glycan with outer Gal | GlcNAc | Vector |  |
| PTL-I | GalNAc, GalNAcα-1,3Gal, GalNAcα-1,3Galβ-1,3/4Glc | GalNAc | Vector |  |
| SJA | Terminal in GalNAc and Gal, anti-A and anti-B human blood group | GalNAc | Vector |  |
| PNA | Galβ1-3GalNAcα-Ser/Thr(T) | Galactose | Vector |  |
| EEL | Galα1-3(Fucα1-2)Gal (blood group B antigen) | Galactose | Vector |  |
| AAL | Fucα1-6 GlcNAc(core fucose), Fucα1-3(Galβ1-4)GlcNAc | Fucose | Vector |  |
| LTL | Fucα1-2Galβ1-4GlcNAc, Fucα1-3(Galβ1-4)GlcNAc, anti-H blood group specificity | Fucose | Vector |  |
| MPL | Galβ1-3GalNAc, GalNAc | GalNAc | Vector |  |
| LEL | (GlcNAc)n, high mannose-type N-glycans | LacNAc | Vector |  |
| GSL-I | αGalNAc, αGal, anti-A and B | GalNAc | Vector |  |
| DBA | αGalNAc, Tn antigen, GalNAcα1-3((Fucα1-2))Gal (blood group A antigen) | GalNAc | Vector |  |
| LCA | α-D-Man, Fucα-1,6GlcNAc, α-D-Glc | Mannose | Vector |  |
| STL | trimers and tetramers of GlcNAc, core (GlcNAc) of N-glycan, oligosaccharide containing GlcNAc and MurNAc | GlcNAc | Vector |  |
| PTL-II | Gal, blood group H, T-antigen | Galactose | Vector |  |
| DSA | β-D-GlcNA, (GlcNAcβ1-4)n, Galβ1-4GlcNAc | GlcNAc | Vector |  |
| VVA | terminal GalNAc, GalNAcα-Ser/Thr(Tn), GalNAcα1-3Gal | GalNAc | Vector |  |
| MAL-I | Galβ-1,4GlcNAc, Siaα2-3Gal, Galβ1-3GlcNAc, Siaα2-3 | Galactose | Vector |  |
| GNA | High-Mannose, Manα1-3Man | Mannose | Vector |  |
| NPL | High-Mannose, Manα1-6Man | Mannose | Vector |  |
| ACA | Galβ1-3GalNAcα-Ser/Thr (T antigen), sialyl-T(ST) tissue staining patterns are markedly different than those obtained with either PNA or Jacalin | Galactose | Vector |  |
| BPL | Galβ1-3GalNAc, Terminal GalNAc | Galactose | Vector |  |
| PHA-E+L | Bisecting GlcNAc, bi-antennary N-glycans, tri- and tetra-antennary complex-type N-glycan | GlcNAc | Vector |  |
| SNA | Sia2-6Gal/GalNAc | GlcNAc | Vector |  |
| RCA120 | β-Gal, Galβ-1,4GlcNAc (type II), Galβ1-3GlcNAc (type I) | Galactose | Sigma-Aldrich |  |
| BS-I | α-Gal, α-GalNAc, Galα-1,3Gal, Galα-1,6Glc | Galactose | Sigma-Aldrich |  |
| PSA | α-D-Man, Fucα-1,6GlcNAc, α-D-Glc | Fucose | Sigma-Aldrich |  |
| SBA | α- or β-linked terminal GalNAc, (GalNAc)n, GalNAcα1-3Gal, blood-group A | GalNAc | Sigma-Aldrich |  |
| WGA | Multivalent Sia and (GlcNAc)_n_ | GlcNAc | Sigma-Aldrich |  |
| UEA-I | Fucα1-2Galβ1-4Glc(NAc) | Fucose | Sigma-Aldrich |  |
| PWM | Branched (LacNAc)_n_ | GlcNAc | Sigma-Aldrich |  |
| ConA | High-Mannose, Manα1-6(Manα1-3)Man, terminal GlcNAc | Mannose | Calbiochem |  |
